# Extranodal NK/T cell lymphoma and lymphomatoid granulomatosis in a patient with chronic lymphocytic leukaemia: Case report for a new perspective on Richter syndrome

**DOI:** 10.1097/MD.0000000000020106

**Published:** 2020-05-08

**Authors:** Jad Abi-Rafeh, Ian V. Beamish, David G. Haegert, Denis Cournoyer, René P. Michel

**Affiliations:** a Faculty of Medicine; b Department of Pathology; c Division of Hematology, Department of Medicine, McGill University, Montreal, Quebec, Canada.

**Keywords:** lymphoma, lymphomatoid granulomatosis, NK/T-cell lymphoma, Richter syndrome, small lymphocytic lymphoma

## Abstract

**Rationale:**

Richter syndrome (RS) defines the transformation of chronic lymphocytic leukemia (CLL) into a more aggressive lymphoma. Although the term RS is most often reserved for transformation of CLL into diffuse large B-cell lymphoma (DLBCL), and less frequently Hodgkin lymphoma , the list of cases with more variable presentations in the literature is growing.

**Patient concerns:**

A 71-year-old Caucasian man initially consulted an otolaryngologist for a 1-year history of nasal congestion.

**Diagnoses:**

The asynchronous occurrence of 2 rare angiocentric Epstein–Barr virus (EBV)-related lymphoproliferative disorders in a patient with CLL, specifically clonally related lymphomatoid granulomatosis (LYG), and an extranodal NK/T-cell lymphoma, nasal type, are described herein.

**Interventions:**

Radiation therapy and a regimen of cis-platinum were administered for the NK/T cell lymphoma, and ibrutinib for LYG.

**Outcomes:**

The patient remains in complete clinical remission 8 years after the diagnosis of chronic lymphocytic leukemia/small lymphocytic lymphoma and recurrent extranodal NK/T cell lymphoma, and 2 years after the diagnosis of clonally related LYG.

**Lessons:**

Although the precise pathogenesis of RS remains incompletely understood, various molecular alterations, in particular long-term immunosuppression, may lead to RS, similar to the causal link existing between non-Hodgkin lymphomas and HIV infection, and post-transplantation lymphoproliferative disorders. EBV infection is linked to the pathogenesis of several types of lymphomas and found in a subset of patients with RS; immunosuppression, in the context of CLL or other pathological conditions or pharmacological agents, can disrupt the fine balance between virus and the host immune system, and result in EBV-driven lymphoproliferations of B-, T-, or NK-cell origin. The findings of our literature review thus suggest that such non-diffuse large B-cell lymphoma , non-Hodgkin lymphoma CLL transformations, may be considered as rare variants of RS.

## 1 Introduction

Richter syndrome (RS) was first reported by Maurice Richter^[1]^ in 1928, describing the appearance of a “reticular cell sarcoma” in a patient with chronic lymphocytic leukemia (CLL). The actual term RS was coined by Lortholary et al^[2]^ in 1964 to describe specifically the transformation of chronic lymphocytic leukemia/small lymphocytic lymphoma (CLL/SLL) into a more aggressive lymphoma. The prevalence of RS among CLL patients ranges from 1% to 25%, with an annual incidence of about 0.5% per year; median time to occurrence of RS from the time of CLL diagnosis ranges from 1.8 to 5 years.^[3–5]^ Prognosis is generally poor with the clonal relationship of RS to the CLL described as key prognostic indicator.^[6]^

The etiology and pathogenesis of RS remain incompletely understood, although the role of immunosuppression and immune dysregulation have been well documented.^[7]^ Infection with Epstein–Barr virus (EBV) is associated with the development of many lymphomas including Burkitt lymphoma, T- and NK-cell lymphomas, post-transplant lymphoproliferative disorders, and Hodgkin lymphoma.^[7]^ Because memory B-cells are a reservoir for EBV in healthy individuals, it is perhaps not surprising that up to 16% of cases of RS may be EBV-positive, underlining the oncogenicity of latent EBV infection in immunocompromised hosts.^[8–10]^ The most common form of RS is transformation to diffuse large B-cell lymphoma (DLBCL) in over 95% of cases and much less frequently (<1%) to Hodgkin lymphoma (HL).^[11]^ Rarely, CLL may co-present with, or transform into other aggressive lymphoproliferative disorders.^[6,11,12]^ Herein, we report the asynchronous occurrence of 2 rare angiocentric EBV-related lymphoproliferative disorders in a patient with CLL, specifically lymphomatoid granulomatosis (LYG) clonally related to the CLL and responsive to ibrutinib, and extranodal NK/T-cell lymphoma, nasal type.

## 2 Case

**Figure 1:**
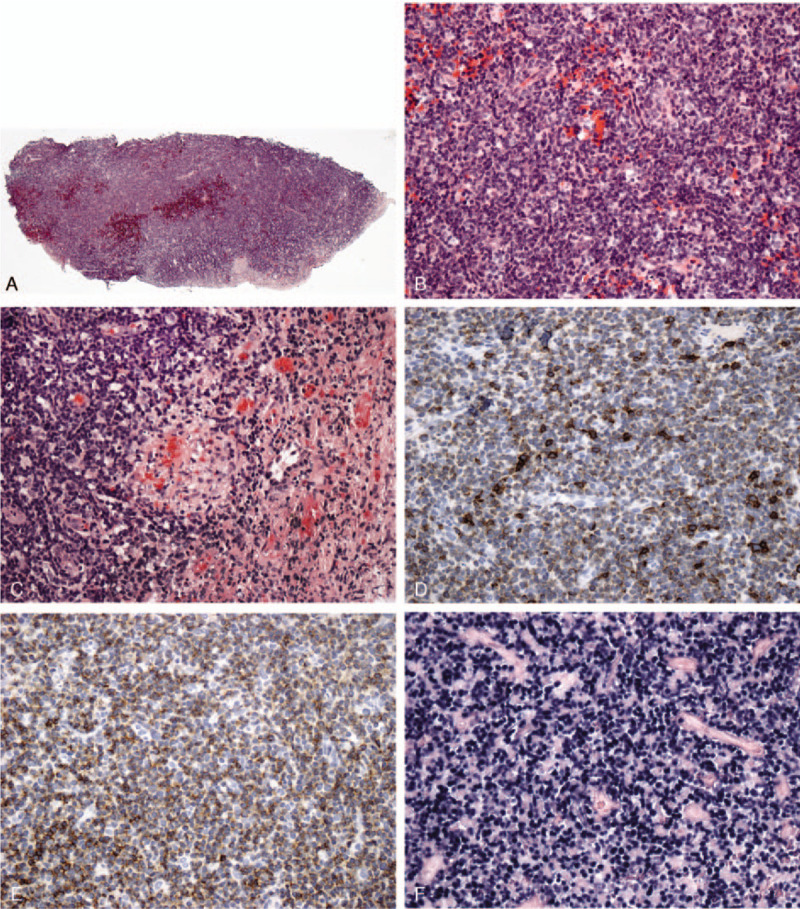
Nasal biopsy of the NK/T-cell lymphoma. A and B. Low and medium power photomicrographs of the biopsy showing replacement by uniform monotonous small to medium lymphoid cells. C. Focal area of the biopsy with small artery invaded by lymphoma cells, that is, angioinvasion (center), and necrosis on the right. D and E. Immunostains for CD3 and CD56, respectively. F. In situ hybridization for Epstein-Barr encoding region positive in all cells.

**Figure 2:**
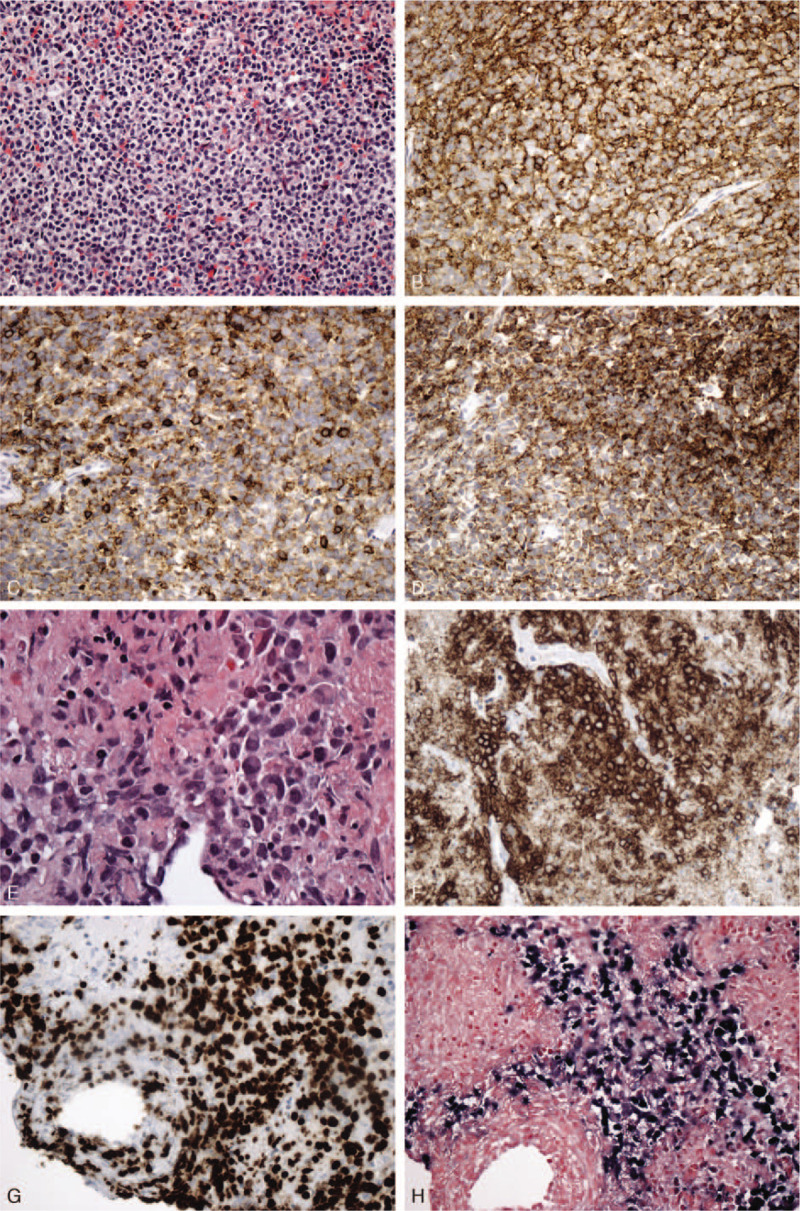
A to D. Chronic lymphocytic leukemia/small lymphocytic lymphoma in core biopsy of the spleen. A. Medium power showing uniform population of small regular lymphocytes. No definite proliferation centers were seen. B to D. Immunostains for CD20 (B), CD5 (C), and CD23 (D) were positive in the vast majority of cells. E to H. Lung biopsy with lymphomatoid granulomatosis. E. High power of an aggregate of the large atypical cells that are positive for CD20 (F), with a high Ki67 proliferation rate (G) and are Epstein-Barr encoding region -positive by in situ hybridization (H). Latter 3 stains at magnification of 200 X.

In November 2011, a 71-year-old Caucasian man consulted an otolaryngologist for a 1-year history of nasal congestion. Nasal endoscopic biopsy of a soft sessile polypoid mass revealed surface ulceration and underlying diffuse infiltration by small to medium lymphocytes with mildly pleomorphic nuclei and inconspicuous nucleoli, invading small vessels with fibrinoid necrosis (Fig. 1A). By immunohistochemistry the cells were positive for CD2, cytoplasmic CD3, CD43, CD56, perforin, and all neoplastic cells were positive for Epstein–Barr encoding region by in situ hybridization (Fig. 1B-F). PCR testing demonstrated no clonal T-cell gene rearrangements; however, clonally rearranged immunoglobulin heavy chain and kappa light chain genes were found indicative of a superimposed B-cell clone. A diagnosis of NK/T-cell lymphoma, nasal type, was made. A CT scan revealed a 3.3 cm mass in the nasal fossa and a 2.3 x 2.3 cm splenic mass; the lungs were not involved. In late December 2011 bone marrow biopsy revealed prominent lymphoid aggregates with the typical immunoprofile of CLL/SLL, EBV-negative, confirmed by flow cytometry; there was no lymphocytosis. In January 2012, a core biopsy of the splenic lesion also revealed involvement by SLL (Fig. 2A-D).

In April 2012, the patient received 54 Gy radiation for the NK/T cell lymphoma. A biopsy of a nasal mass in August 2012 revealed only SLL, EBV-negative, no NK/T cell lymphoma. By November 2012, PET scans demonstrated response of the nasal lesion to radiation, but progression of SLL (still without a component of CLL) in the spleen, liver, and bone, confirmed by bone marrow biopsy; there was no NK/T cell lymphoma. Cytogenetic studies at the time found no deletion of ATM or p53 by fluorescence in-situ hybridization; molecular studies for hypermutation status were not performed. In November 2013, the NK/T cell lymphoma relapsed in the right oropharynx and the patient received radiation with cis-platinum, resulting in remission of this lymphoma. In May 2015, an absolute lymphocytosis of 6.5 x 10^9^/L indicated he had now developed CLL, Rai stage 0. In November 2016, CT scan revealed new bilateral 0.4 cm pulmonary nodules; by February 2017, despite a trial of prednisone, the right lower lobe nodule increased to 1.8 cm. Transthoracic core biopsy revealed subtotal necrosis, foci of preserved large cells expressing CD20 with a high Ki67 proliferation rate, Epstein-Barr encoding region-positive, with admixed T-cells, consistent with a grade 2 to 3 lymphomatoid granulomatosis (Fig. 2E-H). The cells were negative for CD2, CD3, and CD56, thus eliminating a possible NK/T cell lymphoma. Molecular studies revealed a population of clonal B-cells for all strategies, harboring the same clone as the CLL/SLL, with absence of a T-cell clone.

By June 2017, the CLL had progressed to Rai stage II, and because the patient became dyspneic, daily ibrutinib 420 mg was started to treat both the CLL, which had not been previously treated, and the LYG, resulting in resolution of the lung nodules and a reduction in the splenomegaly. In view of the excellent response, ibrutinib was continued, and the patient remains in complete clinical remission 8 years after the diagnosis of CLL/SLL and recurrent extranodal NK/T cell lymphoma, and 2 years after the diagnosis of LYG.

## 3 Discussion

CLL, defined as a neoplasm of small mature B-cells co-expressing CD5 and CD23 with a monoclonal B-cell count ≥5 x 10^9^/L, is the most frequent leukemia in western countries.^[6]^ Its course is variable and 5-year survival rates range from 25% to 95%.^[12]^ Richter syndrome, the transformation of CLL into an aggressive lymphoma, is characterized by the sudden onset of symptoms such as fever, weight loss, rapid lymph nodal enlargement, and variably elevated serum lactate dehydrogenase (LDH) levels.^[13]^ In approximately 80% of patients, the 2 malignancies are clonally related, constituting a “true” transformation; in the remaining 20%, they are unrelated, thereby representing “de novo RS”.^[4,5,14,15]^ Although extranodal involvement is rare, several articles in the literature report gastrointestinal, central nervous system, skin, eye, testicular, pulmonary, and renal lesions.^[3]^

LYG is “an angiocentric and angiodestructive lymphoproliferative disease involving extranodal sites, composed of EBV-positive B cells admixed with reactive T cells, which usually predominate’.^[6]^ Median survival is 4 years and clinical aggressiveness is linked to the proportion of large cells in the lesion.^[16]^ Because LYG in our patient was clonally related to the CLL, we reason that ours is the second reported case of LYG presenting as RS (Table 1).^[17]^ Interestingly, our patient also presented with an extranodal NK/T cell lymphoma, nasal type, another aggressive lymphoma that we believe is an additional rare presentation of RS in the same patient. Although one might argue that our patient had an EBV-positive DLBCL, we favored the diagnosis of LYG primarily because of the exclusive pulmonary localization, the presence of reactive T-cells admixed with the neoplastic B-cells, and, to a lesser extent, because of the patient's ethnic background, the putative immunosuppression produced by the CLL/SLL and its therapies, and the good response to ibrutinib, somewhat supporting the notion that LYG, being of lower grade, has a better prognosis than EBV+ DLBCL.^[18]^

**Table 1:**
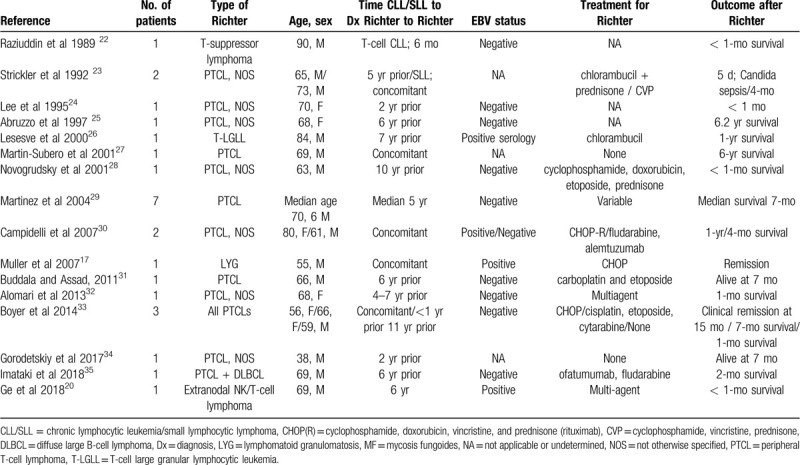
Review of cases of Richter syndrome presenting as rare non-DLBCL, non-Hodgkin lymphoma variants.

Nasal extranodal NK/T cell lymphoma is characterized by angiodestuction, fibrinoid necrosis, expression of cytotoxic molecules and associated with EBV.^[9]^ Patients usually present with upper aerodigestive tract complaints of nasal obstruction, discharge, and epistaxis. Prognosis varies, with some patients responding to therapy, others, especially with disseminated disease, responding poorly despite aggressive regimens.^[9]^ Both EBV and immune dysregulation are described in its pathogenesis, for example, in the context of post-transplant immunosuppression.^[9,19]^ There has only been 1 report by Ge et al^[20]^ describing an NK/T cell lymphoma arising as RS in a patient with CLL, and a small number of other articles describing high-grade T cell-lymphoma transformations in the context of CLL (Table 1).

Although the mechanisms at play in the pathogenesis of RS remain incompletely understood, recent endeavors may explain the evolution of RS clonally related to CLL: Gonzlez-Rincon et al^[21]^ propose both a linear sequence of acquisition of new mutational events in a single clone, and a branched evolution, spatially and temporally heterogeneous, with the appearance of 2 or more tumoral clones, co-existing and evolving in parallel. In contrast, the clonally unrelated cases of RS may be explained by the immunosuppression caused by CLL/SLL and/or its therapy, analogous to the causal link between non-Hodgkin lymphoma and HIV infection and post-transplantation lymphoproliferative disorders.^[7]^ It is therefore unsurprising that in 20% of RS cases, the second neoplasm is not clonally related to the original CLL.^[4,5]^ EBV infection is linked to the pathogenesis of several types of lymphoma and found in a subset of patients with RS.^[8]^ Approximately 95% of the world's population are asymptomatic life-long carriers of EBV, with memory B-cells acting as reservoirs in healthy individuals. Immunosuppression, in the context of CLL, of other pathological conditions or pharmacological agents, can disrupt the fine balance between virus and the host immune system, and result in EBV-driven lymphoproliferations of B-, T-, and NK-cell origin.^[7]^

In summary, we describe herein the case of a patient with simultaneous presentation of CLL and EBV-positive extranodal NK/T cell lymphoma, nasal type, and a concomitant clonally related EBV-positive LYG, and conclude that the development of these 2 rare, extranodal aggressive EBV-associated lymphomas fit the current definition of RS and should thus be classified as such. Finally, we propose that such rare, non-DLBCL, non-HL transformations in CLL patients be considered as “rare RS variants”, and perhaps thus reported on and classified as such in the literature.

Note: The case report which forms part of this work was conducted with informed written consent from the patient. The conduct of this project was also submitted to our institutional Research Ethics Board (REB) which determined that this work was exempt from REB review according to institutional policies.

## Author contributions

**Data curation:** David G. Haegert, Denis Cournoyer.

**Investigation:** Denis Cournoyer.

**Methodology:** David G. Haegert.

**Validation:** Jad Abi-Rafeh.

**Writing – original draft:** Jad Abi-Rafeh, Ian V. Beamish.

**Writing – review & editing:** David G. Haegert, Denis Cournoyer.
